# Very-Brief, Web-Based Interventions for Reducing Alcohol Use and Related Problems among College Students: A Review

**DOI:** 10.3389/fpsyt.2015.00129

**Published:** 2015-09-28

**Authors:** Robert F. Leeman, Elliottnell Perez, Christine Nogueira, Kelly S. DeMartini

**Affiliations:** ^1^Department of Psychiatry, Yale School of Medicine, New Haven, CT, USA; ^2^Department of Psychology, Southern Connecticut State University, New Haven, CT, USA

**Keywords:** young adult, computer, prevention, effectiveness, technology, motivational interviewing, youth, intervention

## Abstract

Very-brief, web-based alcohol interventions have great potential due to their convenience, ease of dissemination, and college students’ stated preference for this intervention modality. To address the efficacy of these interventions, we conducted a review of the literature to identify randomized controlled trials (RCTs). Fifteen published reports were included. All RCTs meeting criteria for inclusion tested an intervention that featured personalized feedback on students’ patterns of alcohol consumption. This review found some evidence to support the efficacy of very-brief, web-based interventions among college students for alcohol use reduction. Several trials, however, reported no evidence of efficacy and the methods of multiple trials raised concerns about potential bias that may have influenced study results. By contrast, this review did not yield evidence to support the efficacy of very-brief, web-based interventions for reduction of alcohol-­related problems among college students. We found evidence to support the efficacy of two main types of intervention content: (a) focused solely on personalized normative feedback designed to correct misconceptions about peer alcohol consumption and (b) multi-component interventions. Future research is needed to test enhancements to very-brief, web-based interventions that feature personalized feedback on patterns of alcohol use and to determine for which types of college drinkers (e.g., heavier or lighter drinkers) these interventions are most efficacious. In addition, future studies are needed to test novel, very-brief, web-based interventions featuring approaches other than personalized feedback. In summary, this review yielded some evidence supporting very-brief, web-based interventions in reducing alcohol use but not related problems in college students. Very-brief, web-based interventions are worth pursuing given their convenience, privacy, and potential public health benefit.

## Introduction

College student heavy drinking is a public health concern. Over 40% of full-time students report at least one past month heavy drinking day (1). Heavy drinking rates are higher among full-time college students than same-aged, non-students (1), suggesting that aspects of the college experience, in particular, appear to increase the likelihood of heavy drinking. Young adult heavy drinking is related to negative consequences, including increased likelihood of poor academic performance, vehicular accidents, injuries, physical fights, risky sexual acts, and sexual assaults (1, 2). While many students “mature out” and reduce heavy use normatively in the years after graduation, a substantial subset continue problematic use (3).

College students tend to have limited motivation to change their drinking behavior (4, 5) and accordingly, rarely seek specialized services (6). Because brief motivational interventions are designed to increase motivation to change, they are well-suited to college drinkers (7). Meta-analyses and narrative reviews (8–14) have found that brief interventions are associated with significant reductions in college student drinking up to 6 months later, though effect sizes associated with these interventions tend to be small (15, 16).

Unfortunately, not enough students have access to efficacious brief interventions. Only about 50% of U.S. colleges make empirically supported interventions available (17, 18). Making interventions available via computer, along with making them as brief as possible, will facilitate dissemination to the greatest number of students.

Computer-based interventions have pragmatic advantages, including standardization, time, and cost-effectiveness (9, 10, 19–21), and college students prefer them (5, 6). Meta-analyses support their efficacy, including those with and without direct participant/researcher contact (9, 22). A recent meta-analysis found that face-to-face and computer-based alcohol interventions had comparable evidence for efficacy in the short-term and for four out of the five long-term outcomes that were examined (10). Face-to-face interventions have certain advantages over computer-based interventions (23), but they are relatively costly and time-intensive (10, 18). Accordingly, the authors of the aforementioned meta-analysis argued that computer-based approaches are beneficial for early intervention (10).

Web-based interventions are a subset of computer-based interventions that require no actual participant/researcher contact (24–27). Web-based interventions are particularly convenient and private (24, 26, 28) though the lack of participant/researcher contact raises some concerns. For instance, some participants may complete a web-based intervention while distracted (by other people, television, etc.), whereas these distractions can be reduced or eliminated when utilizing a standardized setting (e.g., student health service, office). Nonetheless, early evidence suggests that web-based interventions are efficacious (28), including among young adult drinkers (29), though there have been relatively few controlled studies in this population (26).

To ensure that the present review specifically tested web-based interventions, we excluded studies with research designs that entailed direct, face-to-face contact between researchers and participants, even if the tested computer-based intervention was fully automated and did not require this interaction. A recent study made the same distinction, comparing three randomized controlled trials (RCTs) of *computer*-based alcohol reduction interventions administered in-person with three RCTs testing *web*-based interventions without direct researcher/participant contact (27). The distinction between computer- and web-based interventions is important, because while both types of intervention had evidence for efficacy, the computer-based interventions had stronger empirical support than the web-based interventions. This study focused on one particular type of brief intervention: provision of only personalized normative feedback that compared the student’s self-reported drinking with average student drinking levels and the student’s perceptions of these drinking levels in order to correct overestimations of peer drinking (26, 30–33). Thus, it is unclear whether or not other types of web-based alcohol intervention may have efficacy comparable to in-person, computer-based administration. Given the potential advantages of web-based interventions to colleges and universities in minimizing cost and time expenditure while maximizing dissemination, it is important to assess thoroughly the efficacy of these programs.

In addition to the question of whether interventions can be efficacious in absence of in-person contact, intervention duration is another important practical issue. Very-brief interventions may have efficacy similar to longer interventions and lead to even wider dissemination (10, 34). In three recent RCTs, brief (40–60 min) and very-brief (≤10–15 min) interventions were compared with no significant differences in outcomes among college students (20, 30, 35). College administrators and clinicians are concerned about other negative health behaviors among their students, including drug use, cigarette smoking, risky sexual behavior, and eating disorders (36–39). Very-brief alcohol screening and intervention will allow colleges more time to address these other behaviors as well. Considering the short duration, convenience, and remote chance of adverse events associated with very-brief, web-based interventions, even small effect size reductions in alcohol consumption or related problems would make these programs worthwhile.

In summary, evidence suggests that brief interventions can reduce drinking, yet many colleges do not provide these interventions. Very-brief, computer-based programs (particularly web-based, which require no direct contact with students/participants) have the potential to increase student access to alcohol reduction interventions dramatically. The goal of the present review is to evaluate the efficacy of very-brief, web-based alcohol reduction interventions for college students. For this review, very-brief was defined as requiring no more than 15 min for participants both to complete assessments required for the generation of intervention content and to review the intervention content itself. Defining very-brief as entailing an average of no more than 15 min has precedent in the literature (20, 35). While there have been multiple reviews of brief interventions for college students (8, 11–14, 16), including reviews focused on computer- and web-based interventions (9, 10, 29), to our knowledge, this is the first review focusing specifically on the efficacy of very-brief, web-based alcohol reduction interventions for college students.

Although meta-analysis of this area would be valuable, it was premature at this time due to the relatively small number of eligible studies our review yielded; the heterogeneity of methods across these studies; and differences in intervention content (described below). We expected that these key differences across studies would be associated with disparities in the true effect sizes of these studies. Therefore, a meta-analysis would require a random-effects model (40). According to Borenstein et al. [(40), p. 363], when a random-effects model is based on a small number of studies, the “estimate of the between-studies variance (T^2^) may be substantially in error,” and therefore “not only is the point estimate likely to be wrong but the confidence interval may provide a false sense of security.” Taking these factors into consideration, we opted not to conduct a meta-analysis, however between-groups effect sizes were reported for each outcome in each individual study included in the review.

## Materials and Methods

**Figure 1:**
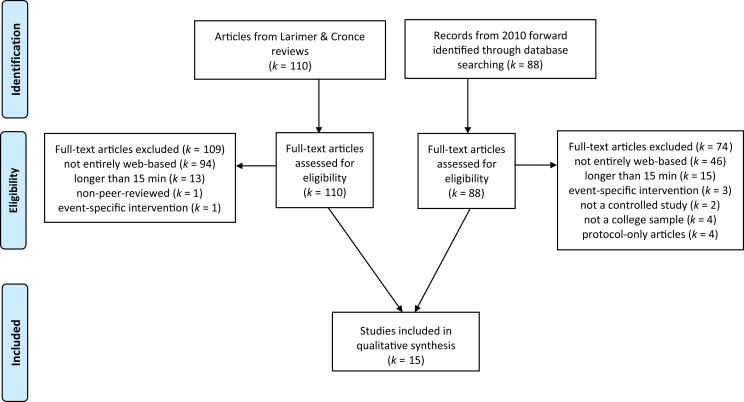
**Study flow diagram**.

Larimer and Cronce’s (11, 12, 41) series of reviews of individual alcohol interventions for college students were used as the basis for the current review. Larimer and Cronce’s procedure involved searching multiple electronic databases, including PsycInfo and MEDLINE, using an exhaustive set of keywords [see Ref. (12)] for a detailed list of databases and keywords). Their reviews included only reports of RCTs (i.e., individuals or groups randomly assigned to one of two or more conditions consisting of at least one active intervention condition and at least one control condition). The full texts of all 110 articles included in their reviews were considered for inclusion in the present review. Furthermore, we utilized Larimer and Cronce’s search strategy to update the present review to include papers published since their most recent literature search ended in early 2010 through September 2014. Based on reviews of article titles and abstracts, those that were clearly not RCTs (e.g., reviews of the literature/meta-analyses, surveys) were eliminated from further consideration. The full-text of each of the remaining articles was then evaluated by two raters using the criteria below to isolate published studies that rigorously tested very-brief, web-based alcohol reduction interventions among samples of college students (Figure 1). Given that our goals were more specific than those of Larimer and Cronce, we used a more specific set of inclusion criteria. Any disagreements between the raters were resolved based on discussion. Criteria for inclusion in the present review were considered in the following order:

1. Data collection must have been entirely web-based (including no non-web, computer-based programs, e.g., on CD-ROM) with no direct contact between investigators and participants, though in-person recruitment activities prior to actual study participation (e.g., recruitment talks in course meetings) and non-web-based means of reminding participants to complete assessments (e.g., phone calls) were allowed.
2. Assessments that contributed to intervention materials (e.g., personalized feedback form) and time for participants to consider the intervention materials must have taken no longer than 15 min. Determination of intervention duration was based either on reported average time of completion in published papers or by contacting investigators for this information. In absence of data on average time to complete, we requested that investigators estimate the average amount of time the intervention itself required. Investigator use of additional questions/measures that did not contribute to the intervention component was allowed, as these additional measures could theoretically be removed, leaving a very-brief, web-based intervention. In some cases, clarification was sought from investigators to make this distinction.
3. The intervention must have concerned alcohol use in general, not event-specific drinking (e.g., spring break, 21st birthday drinking). These targeted interventions are valuable but their utility is limited to students in these particular situations. Our interest was in interventions targeting general patterns of alcohol consumption that would have broad dissemination potential.
4. The intervention tested had to be focused primarily on alcohol consumption.
5. The study sample had to be composed primarily of college students.
6. Studies must have utilized random assignment to study conditions on an individual basis, thus randomization could not be by class, course, college/university, etc.
7. Results must have been published as a fully peer-reviewed report (i.e., no conference abstracts or reports from conference symposia).

We conducted a risk of bias assessment using the Cochrane Handbook (42) as a guide. All individual articles included in the review were evaluated with respect to the following criteria: (1) selection bias: whether there were effective procedures to assign participants randomly to study conditions and whether steps were in place to conceal which condition assignments were upcoming in sequence; (2) performance bias: whether both investigators and participants were blinded to study condition; (3) attrition bias: whether outcome data were incomplete due to participants not remaining in the study and whether small-to-moderate amounts of missing data were accounted for analytically; and (4) reporting bias: whether complete outcome data (i.e., all outcomes stipulated *a priori*) were reported. Detection bias (i.e., whether outcome assessors are blinded to study hypotheses and condition assignments) is another criterion typically evaluated, however this potential bias is not relevant to web-based studies, given that all data collection occurs via the web and thus is not administered by a study investigator. Also given the lack of direct contact between investigators and participants in web-based studies, we focused primarily on participant blinding when evaluating performance bias. The risk of bias assessment was conducted by two separate raters with any disagreements resolved through discussion.

Two general types of alcohol-related outcomes were considered: all available measures of alcohol consumption and of alcohol-related problems. Regarding related problems [e.g., Ref. (43, 44)], we considered all pertinent constructs, including measures of alcohol-related consequences and measures indicative of risk of alcohol use disorders, such as the Alcohol Use Disorders Identification Test [AUDIT; (45)] and the CAGE questionnaire (46). Outcome data for all eligible studies were extracted from published papers by two raters with any disagreements resolved through discussion. In our reports of results, we focused on primary outcomes as opposed to subgroup analyses.

## Results

### Overview

After removing reports from surveys and reviews of the literature, the updated review procedure yielded 88 studies published between early 2010 and September 2014. These studies were evaluated for inclusion in the present review, along with the 110 studies included in the three reviews by Larimer and Cronce for a total of 198 studies (Figure 1). Of these, the 15 studies reported in Table 1 met criteria for inclusion in the present review.

**Table 1:** **Published reports of controlled studies of very-brief (≤15 min) web-based interventions for college students/young adults**.

References	Sample	Elements	Auto fb?	Multi fb?	*N*	pct. Male (%)	*M* age	Control group	Time-points (follow-up rates)	Effect size estimates for alcohol-consumption and alcohol-related problems outcomes
Bewick (19)	All students	F S R I	Yes	Yes	506	31	21	Assess only	12 weeks (63%)	Alcohol units per occasion: 0.29 Units of alcohol per week: 0.04^a^ Proportion heavy drinking pre/post: 0.17^a^ CAGE measure: 0.02^a^
Bewick (47)	Any drinkers	F S R I	Yes	Yes	1112	27	21	Assess only	8 weeks (62%) 16 weeks (42%) 24 weeks (34%)	Units of alcohol per occasion Immediate versus delayed: −0.02 (8 weeks), −0.09 (16 weeks), −0.05 (24 weeks)^a^ Immediate versus Control: −0.01 (8 weeks), −0.01 (16 weeks), −0.02 (24 weeks)^a^ Alcohol units per week Comparison between immediate versus delayed intervention conditions: –0.09 (8 weeks), −0.10 (16 weeks), −0.13 (24 weeks)^a^ Immediate versus control condition: −0.09 (8 weeks), −0.02 (16 weeks), 0.01 (24 weeks)^a^
Bewick (48)	Any drinkers	F S R I	Yes	Yes	1478	30	21	Assess only	16 weeks (50%) 34 weeks (44%)	Units of alcohol per occasion: 0.36 (16 weeks), 0.20 (34 weeks)^a^ Units of alcohol per week: 0.11 (16 weeks), −0.02 (34 weeks)^a^
Cunningham (24)	Heavy drinkers	F S I	Yes	Yes	425	53	23	Assess only	6 weeks (68%)	AUDIT-C scores (0.13)^a^
Ekman (49)	Heavy drinkers	F S I	Yes	Yes	654, but results only for those with full data: *n* = 158	42	18–20 (16%) 21–25 (76%) ≥26 (8%)	Very-brief feedback	3 months (38%) 6 months (24%)	Weekly consumption in grams: 0.19 (3 months), 0.23 (6 months)^a^ Peak eBAC: −0.11 (3 months), −0.05 (6 months)^a^ Heavy drinking: 0.04 (3 months), 0.13 (6 months)^a^
Kypri (50)	Hazardous drinkers	F S R I	Yes	Yes	2435	55	20	Assess only	1 month (78%) 6 months (65%)	Quantity per occasion: 0.11 (1 month), 0.05 (6 months)^a^ Overall volume of alcohol consumption: 0.15 (1 month), 0.13 (6 months)^a^ Frequency of alcohol consumption: 0.17 (1 month), 0.15 (6 months)^a^ Binge drinking: 0.14 (1 month), 0.06 (6 months)^a^ Heavy drinking: 0.38 (1 month), 0.33 (6 months)^a^ Academic consequences: 0.08 (1 month), 0.04 (6 months)^a^ Other consequences: 0.05 (1 month), 0.05 (6 months)^a^
Kypri (51)	Heavy drinkers	F S R I	Yes	Yes	1789	35	20	Assess only	5 months (79%)	Quantity per occasion: 0.09 Overall volume of alc. cons.: 0.16 Frequency of alcohol consumption: 0.13 Binge drinking: 0.12^a^ Heavy drinking: 0.24^a^ Academic consequences: 0.13
Kypri (52)	Heavy drinkers	F S R I	Yes	Yes	3422	43	20	Assess only	5 months (83%)	Quantity per occasion: 0.04^a^ Overall volume of alc. cons.: 0.00^a^ Frequency of alcohol consumption: 0.03^a^ Binge drinking: 0.10^a^ Heavy drinking: 0.14^a^ Academic consequences: 0.01
LaBrie (30)	Heavy drinkers	F	Yes	No	1480^b^	43^c^	20^c^	Attention control	1 month (90%) 3 months (87%) 6 months (84%) 12 months (86%)	Drinks per week:0.19 (1 month), 0.24 (3 months), 0.20 (6 months), 0.13 (12 months)^a^,^d^ Frequency of alcohol cons.: 0.24 (1 month), 0.18 (3 months), 0.28 (6 months), 0.12 (12 months)^a^,^d^ Peak number of drinks: 0.02 (1 month), 0.07 (3 months), 0.11 (6 months), −0.01 (12 months)^a^,^d^ Negative consequences: 0.19 (1 month), 0.20 (3 months), 0.24 (6 months), 0.18 (12 months)^a^,^d^
Lewis (31)	Heavy drinkers	F	Yes	No	240^e^	42^f^	20^f^	Attention control	3 months (90%)	Drinks per occasion: 0.43 (3 months), 0.34 (6 months)^a^ Drinks per week: 0.28 (3 months), 0.18 (6 months)^a^
									6 months (85%)	Frequency of alcohol consumption:0.32 (3 months), 0.14 (6 months)^a^ Negative conseq.: 0.13 (3 months), −0.05 (6 months)^a^
Martens (53)	Varsity athletes	F S	No	Yes	263	24	20	Educ only	1 month (89%) 6 months (81%)	Drinks per week: *n*^2^ = 0.004 (1 month), 0.005 (6 months) Peak eBAC: *n*^2^ = 0.007 (1 month), 0.04 (6 months) Alc.-related probs: *n*^2^ = 0.017 (1 month), 0.005 (6 months)
McCambridge (54)	All students	F S I	Yes	Yes	7809	49	18–20 (27%) 21–25 (56%) ≥26 (17%)	Assess only and no contact	3 months (52%)	Intervention versus. assess only AUDIT-C: −0.01 pct. risky drinking: 0.03^a^ Intervention versus no contact AUDIT-C 0.05 pct. risky drinking: 0.08^a^
Moreira (55)	All students	F R I	No	Yes	1751	38	17–19 (60%) 20–24 (34%) ≥25 (6%)	Assess only and no contact	6 months (50%) 12 months (41%)	Quantity of alcohol per occasion 0.05 (6 months), 0.10 (12 months)^a^ AUDIT score: 0.03 (6 months), 0.12 (12 months)^a^ Alcohol-related problems −0.02 (6 months), −0.03 (12 months)^g^,^a^
Neighbors (26)	Heavy drinkers	F	Yes	No	818	42	18	Attention control	6 months (92%) 12 months (87%) 18 months (84%) 24 months (81%)	Weekly drinking Time × study condition interactions: range from 0.07 to 0.16 Heavy drinking Time × study condition interactions: range from −0.03 to 0.08 Alcohol-related problems Time × study condition interactions: range from −0.02 to 0.11
Palfai (56)	All students	F S R I	Yes	Yes	705	29	18	Non-alcohol-related feedback	5 months (53%)	Drinks per week :0.07^a^ Any drinking: 0.21^a^ Heavy drinking: 0.06^a^ Risky drinking: −0.01^a^ Alcohol-related consequences: −0.11^a^

*All effect size estimates are Cohen’s *d* unless otherwise noted. Elements = intervention elements [F = personalized feedback; S = protective behavioral strategies; R = resources for behavior change (e.g., counseling); I = general alcohol information]; Auto fb? = is feedback generated automatically by program? Multi fb? = does feedback include multiple components? *N* = number of participants; assess = assessment; educ = education; eBAC = estimated blood alcohol concentration; wk = week; mo = month*.

*Alcohol-related sample distinctions: all students = anyone from the student body allowed to enroll; any drinkers = study had a minimal drinking inclusion criterion; heavy drinkers = study required participants to self-report a substantial amount of alcohol consumption to enroll; hazardous drinkers = study required participants to score of 8 or higher on the Alcohol Use Disorders Identification Test (AUDIT) in order to enroll*.

*AUDIT-C: first three items of the AUDIT, which concern alcohol consumption*.

*^a^Effect size calculations made by authors of the present review*.

*^b^One study condition omitted from review because it was not very-brief in duration*.

*^c^Figures reported for the entire sample though we do not consider results with one study condition in the present review because it was not very-brief in duration*.

*^d^For parsimony, only comparison between control condition and personalized normative feedback, including typical student norms reported, because this type of feedback had the strongest evidence of efficacy in this study, which included eight different types of personalized normative feedback*.

*^e^Study conditions focused on alcohol-related risky sexual behavior omitted, leaving only an intervention focused solely on alcohol and the control condition*.

*^f^Figures reported for the entire sample though we do not consider results with two study conditions in the present review because the intervention in these conditions was not focused solely on alcohol*.

*^g^Authors also reported results with higher-risk subsample but results were similar, thus we opted to display only results involving larger sample*.

### Risk of bias assessment

Results of a risk of bias assessment among all studies included in the review are reported in Table 2. In addition to detection bias, reporting bias was not an issue with no studies raising concerns with regard to this criterion. The majority of studies raised low concern regarding selection bias though multiple articles provided insufficient information in order to make an evaluation. While the majority of studies raised low concern regarding performance bias, several studies raised high concern due to a possibility that participants may have been able to determine when they were in the control condition due to aspects of these studies’ procedures. We considered assessment-only control to raise greater concern about performance bias than control conditions in which participants were exposed to alternate information [e.g., a control condition presenting non-personalized educational information on alcohol; (53)] unless considerable efforts were made to obscure the inherent difference in time and effort between conditions. Regarding attrition bias, we considered a high level of attrition (i.e., approximately 50% or higher) to be inherently problematic. For studies with low-to-moderate levels of attrition, we considered risk of bias to be low when attrition levels did not differ considerably between study conditions and when adequate accounting was made analytically for missing data. Several studies raised high concern regarding attrition bias. Overall, 10 of the 15 studies included raised high concern regarding risk of bias on at least one criterion.

**Table 2:** **Risk of bias assessment among studies included in the present review**.

Reference	Selection bias	Performance bias	Detection bias	Attrition bias	Reporting bias
Bewick (19)	Randomization using statistical package feature: low	In addition to general concerns with assessment-only approach, intervention condition received an additional email invitation at Week 6 and open access to intervention site, also greater compensation in intervention group: high	Low	Moderate attrition rate, but equivalent between groups, no analytic strategy for handling missing data: high	All pre-specified outcomes reported: low
Bewick (47)	Lack of information about how randomization was performed: unclear	In addition to general concerns with assessment-only approach, intervention condition had open access to intervention site: high	Low	Moderate attrition rate, higher attrition in intervention groups: high	All pre-specified outcomes reported: low
Bewick (48)	Lack of information about how randomization was performed except that it was performed by someone outside of the study: unclear	In addition to general concerns with assessment-only approach, intervention condition received an additional email invitation at Week 7 and open access to intervention site: high	Low	High attrition rate, assignment to intervention condition was a predictor of dropout: high	All pre-specified outcomes reported: low
Cunningham (24)	Lack of information about how randomization was performed: unclear	Assessment-only control. Before re-contact, potential participants informed that some students would receive additional information about campus drinking: high	Low	Moderate attrition rate but equivalent by group and no relationship been alcohol outcome and dropout: low	Pre-specified outcome reported: low
Ekman (49)	Computer randomization, participants not told there were two types of feedback nor what their condition assignment was low	Control group received a more brief version of personalized feedback: low	Low	Very high attrition rates high	All pre-specified outcomes reported: low
Kypri (50)	Computer-based, automated randomization: low	Assessment-only control, but participants blind to purpose of study. Study presented as a series of surveys. Researchers also blind to group allocation: low	Low	Attrition rate modest at 1 month, moderate at 6 months, equivalent across conditions. Missing data accounted for analytically: low	All pre-specified outcomes reported: low
Kypri (51)	Computer-based, automated randomization: low	Assessment-only control, but participants blind to purpose of study; study presented as a series of surveys, researchers also blind to group allocation: low	Low	Attrition rate modest, equivalent across conditions. Missing data accounted for analytically: low	All pre-specified outcomes reported: low
Kypri (52)	Computer-based, automated randomization: low	Assessment-only control, but participants blind to purpose of study; study presented as a series of surveys, researchers also blind to group allocation: low	Low	Attrition rate low, equivalent across conditions. Missing data accounted for analytically: low	All pre-specified outcomes reported: low
LaBrie (30)	Computer-based, automated randomization: low	Assessment plus attention control providing non-alcohol-related generic feedback regarding campus norms (e.g., frequency of text messaging). Unclear what, if anything, participants were told about condition assignment/provision of additional information: Unclear	Low	Attrition rate low, equivalent across conditions. Missing data accounted for analytically: low	All pre-specified outcomes reported: low
Lewis (31)	Computer-based, automated randomization: low	Participants told they would be randomly assigned and that they may or may not receive information comparing their drinking and/or sexual behavior to other students at the university. Assessment plus attention control providing non-alcohol-related generic feedback regarding campus norms (e.g., frequency of text messaging): high	Low	Attrition rate low, equivalent across conditions. Missing data accounted for analytically: low	All pre-specified outcomes reported: low
Martens (53)	Randomization using random number table, but no further information given: unclear	Assessment plus education control. Feedback produced by research assistant who entered data into program and emailed link containing feedback to participant. Link to control condition provided in similar way: low	Low	Attrition rate low, equivalent across conditions. Missing data accounted for analytically: low	All pre-specified outcomes reported: low
McCambridge (54)	Automated randomization *a priori* before students agreed to participate: low	Assessment-only and no-contact control conditions but participants unaware they were participating in an intervention study and that they had been randomized to a condition. The invitation to participate described a general lifestyle rather than an alcohol study: low	Low	Very high attrition rates though equivalent across conditions: high	All pre-specified outcomes reported: low
Moreira (55)	Randomization via concealed centrally allocated computer-generated random numbers: low	Assessment-only, main control group contacted at two follow-ups, delayed control contacted at only 1. Neither researchers nor participants were aware of condition assignment at time of randomization. Feedback emailed by a research assistant: low	Low	Very high attrition rates though equivalent across conditions: high	All pre-specified outcomes reported: low
Neighbors (26)	Computer-based, automated randomization: low	Participants told they would be randomly assigned and that they may or may not receive information comparing their drinking to other students at the university. Assessment plus attention control providing non-alcohol-related generic feedback regarding campus norms (e.g., pct of students who work): high	Low	Attrition rate low at initial follow-up, low-to-moderate at later follow-ups, equivalent across conditions. Missing data accounted for analytically: low	All pre-specified outcomes reported: low
Palfai (56)	Lack of information about how randomization was performed: unclear	Control condition received assessment plus non-alcohol feedback: low	Low	Very high attrition rates though equivalent across conditions: high	All pre-specified outcomes reported: low

### Outcomes reported and effect size estimates

Given the small number of studies and other factors discussed above, we opted not to conduct a meta-analysis, however between-groups effect sizes for outcomes reported in each study are provided in Table 1. When authors reported conventional between-groups effect size estimates (Cohen’s *d*, *n*^2^), these were repeated in Table 1. When conventional effect sizes were not reported, we calculated Cohen’s *d* for between-groups effects based on means and SDs reported in the papers, or obtained from the authors, or based on test statistics including odds ratios.

When evaluating outcomes from these RCTs, we used Cohen’s (57) suggested benchmarks of *d* = 0.20 or *n*^2^ = 0.02 for a small effect size (from this point forward we will refer to *d* = 0.20 only since only one study reported *n*^2^). Again, we considered even small effect sizes to be valuable given the brief duration, convenience, and low risk associated with the interventions tested in these RCTs. All 15 RCTs reported alcohol consumption outcomes. There was a lack of consistency across trials in terms of which alcohol-related outcomes were reported. A measure of overall drinking (often drink units per week) was reported most commonly (*k* = 12 trials), followed by drinks per occasion (*k* = 10), heavy episodic drinking (*k* = 9), frequency of any drinking (*k* = 7), and peak drinking including peak estimated blood alcohol concentration (eBAC) (*k* = 3).

### Commonalities across individual RCTs

All RCTs tested an intervention that included some form of personalized feedback regarding the student’s alcohol use, though trials differed regarding the type of information provided in this feedback. In most RCTs, personalized feedback was generated automatically by the intervention program, however in two trials (53, 55), some direct effort by study staff was required in order to produce the feedback. Another commonality was that they were recent trials. The earliest paper was published in 2008 (19). Among RCTs that reported it, mean age was relatively similar, with most reporting an average age of 20–21. Two trials enrolled comparably younger samples with a mean age of 18 (26, 56). Women made up the majority of the sample in all but two trials (24, 50).

### Methodological distinctions

Three key methodological details differed across RCTs: sample inclusiveness, type of control/comparison group, and follow-up time-points. Whether studies were inclusive of students in general or focused on at-risk drinkers was a key distinction. The modal approach was to enroll at-risk students only (*k* = 8), typically based on frequency of heavy drinking and/or amount of overall weekly drinking. Other approaches used in more than one trial were to invite students regardless of drinking status (*k* = 4) or to invite drinkers at any level (*k* = 2).

The nature of the control/comparison group was another distinguishing detail. The modal approach was an assessment-only comparison group (*k* = 9). In two of these studies, a second comparison/control group receiving no contact was also included. Other approaches used in more than one study were an attention control condition (*k* = 3) and alternate personalized feedback (*k* = 2), either more brief than in the intervention condition (49) or concerning a topic other than alcohol use (56).

Follow-up time-points also differed. Most studies had their first follow-up a minimum of 3 months post-intervention (*k* = 10). Of the studies that included a follow-up time-point within 3 months of the intervention (*k* = 5), all but one also included one or more longer-term follow-ups.

Initial sample size and follow-up rates also differed across studies. Most of the included RCTs enrolled large numbers of students, with the majority (*k* = 8) enrolling over 1,000 students initially, however there was a substantial range. A study of student athletes (53) enrolling 263 participants was the smallest, though Ekman et al. (49) reported results involving only 158 of the 654 participants they enrolled. Though only 240 participants from a larger-scale study conducted by Lewis et al. (31) were considered for the present review, the larger study contained two other conditions that provided substantial intervention material on a health behavior other than alcohol use (i.e., sexual risk behaviors). A study conducted by McCambridge et al. (54) enrolling 7809 students, regardless of drinking status, was the largest. Follow-up rates also differed across studies. Several studies (*k* = 7) reported follow-up rates in the 70%-range or higher, while a number of studies (*k* = 5) retained in the 50%-range or lower.

### Intervention content

Randomized controlled trials fell into two distinct types regarding intervention content in general and in the content of personalized feedback summarizing students’ patterns of alcohol consumption: (a) those focused solely on personalized normative feedback (*k* = 3) and (b) multi-component interventions (*k* = 12). The former type provided only highly focused personalized, normative feedback based on the student’s own drinking, average drinking levels of other students on campus and the student’s perception of typical drinking in order to correct peer drinking misperceptions (26, 30, 31). This highly focused personalized feedback was the only intervention content provided. Each of the three studies of this type was unique. One trial compared the efficacy of gender-specific versus gender non-specific personalized normative feedback as well as single versus biannual administration (26). The second such trial tested eight different variants of student referents (e.g., gender-, race-, Greek status-specific; typical student) for the personalized normative feedback (30). The most recent of these trials tested personalized normative feedback focused on alcohol only as well as variants focused on both alcohol and related sexual risk (31).

In the second type of study, multiple intervention components were provided, including personalized feedback that addressed multiple aspects of students’ drinking (e.g., estimated monetary expenditure on alcohol, alcohol dependence risk), not only normative drinking information (*k* = 12). Other components included in multi-component interventions were protective behavioral strategies (*k* = 11), which are cognitive-behavioral techniques designed to limit alcohol use (e.g., alternating alcoholic with non-alcoholic beverages; slowing pace of drinking) (58, 59); facts about alcohol (e.g., relevant laws concerning alcohol use and driving under the influence) (*k* = 11); and available resources for students interested in taking action to reduce their alcohol consumption (e.g., counseling resources) (*k* = 8).

### Effect size estimates in general and in relation to distinctions between RCTs

The largest effect sizes were in the small-to-moderate range (57). On the opposite end of the spectrum, two RCTs reported effect sizes in the opposite direction (i.e., comparison group fared slightly better) of 0.10 or more. Nine of the 15 included RCTs reported at least one effect size of *d* = 0.20 or greater for an alcohol use reduction outcome (a total of 12 alcohol reduction outcomes with effect size estimates at this level). The outcomes that were affected positively by the interventions tested varied across trials with three effect sizes of 0.20 or greater each for drinks per occasion, overall drinking, and frequency of any drinking; along with two for heavy drinking and one for peak drinking. By contrast, only 10 of the 15 RCTs reported alcohol-related problems outcomes and among those, only one RCT yielded an effect size estimate of 0.20 or greater (30).

Table 3 includes comparisons between RCTs reporting at least one effect size estimate of 0.20 or greater for an alcohol consumption outcome (*k* = 9) versus those that did not (*k* = 6) based on the distinctions described above: sample inclusiveness (all students versus those including only drinkers); type of comparison group (assessment and/or no contact versus any type of “active” comparison group); time of earliest follow-up assessment (less than 3 months versus 3 months or greater); initial sample size (greater or less than 1,000); sample retention rate at follow-up (70% or greater versus less than 70%); and intervention content (personalized normative feedback only or multi-component intervention). None of these comparisons yielded any study characteristics that were clearly associated with greater likelihood of reporting at least one result with an effect size of 0.20 or greater (Table 3).

**Table 3:** **Number of studies reporting at least one effect size estimate greater than or equal to 0.20 for an alcohol consumption outcome by key study characteristics**.

Sample inclusiveness	All students included (*k* **=** 4)	Only drinkers included (*k* **=** 10)
	2	6
Type of comparison group	Assessment only (*k* = 9)	“Active” control (*k* = 6)
	4	5
First follow-up time-point	<3 months post-intervention (*k* = 5)	≥3 months post-intervention (*k* = 10)
	3	6
Initial sample size	>1,000 (*k* = 8)	<1,000 (*k* = 7)
	4	5
Follow-up retention rate	≥70% (*k* = 7)	<70% (*k* = 8)
	5	4
Intervention content	Personalized normative feedback only (*k* = 3)	Multi-component (*k* = 12)
	2	7
Among multi-component intervention studies (*n* = 12)		
Number of components	All four (*k* = 7)	Less than four (*k* = 5)
	5	2

*“Active” control conditions included attention control, alternate forms of personalized feedback and education. The four intervention components referred to were personalized feedback, protective behavioral strategies, resources for behavior change (e.g., counseling), and general alcohol information*.

Both types of intervention (i.e., personalized normative feedback only and multi-component intervention) were empirically supported. Two out of the three personalized normative feedback only trials reported multiple alcohol outcomes with effect size estimates of *d* = 0.20 or greater (30, 31). Given that the LaBrie et al. (30) trial tested eight variants of student referents for the personalized feedback, for parsimony, we calculated and compared effect size estimates between the control condition and the version reported by the authors as having the strongest supporting evidence (i.e., typical student norms), though results were generally similar across variants. Compared to the control condition, the typical student norms version was associated with reductions in frequency of alcohol use, drinks per week and negative consequences of 0.20 or greater. For the Lewis et al. (31) trial, we considered only personalized normative feedback focused on alcohol (this study also tested feedback on alcohol-related sexual risk). However, the alcohol-focused feedback showed evidence of efficacy with decreases in frequency of alcohol drinking, drinks per occasion, and drinks per week of 0.20 or greater for the intervention compared to control. Neighbors et al. (26) reported an interaction with time for the gender-specific personalized normative feedback with biannual administration in comparison with control that nearly met this threshold (*d* = 0.16).

Seven of the 12 RCTs utilizing a multi-component intervention reported an effect size of 0.20 or greater (19, 48–51, 53, 56). Among these, two interventions stand out as having more than one positive trial (19, 48, 50, 51). Bewick et al. reported reductions in drink units per occasion with effects greater than 0.20 in two trials: one in which all students were invited to participate (2008) and in a subsequent trial enrolling only drinkers (2013). A 2010 study in which only drinkers were enrolled did not yield any outcomes with effect sizes of 0.20 or greater (47). Kypri et al. reported reductions in heavy drinking with effects greater than *d* = 0.20 in two trials, both of which enrolled at-risk drinkers only. Two large RCTs conducted by Kypri et al. (51, 52) were conducted in parallel, one recruiting only students from a particularly high-risk indigenous population (2013) and the second recruiting among all other students (2014). Other than the sampling method, all procedures were the same, yet these were separate trials and are reported as such in Table 1. The intervention had stronger evidence in the trial enrolling members of the indigenous population than in the trial enrolling a general at-risk drinking sample (Table 1). A study reported by Ekman et al. (49) yielded evidence of a greater reduction in weekly alcohol consumption in the intervention compared with the control group among heavy drinking students, however they reported only results among the small subset of the sample with complete data (*n* = 154 compared to full *N* of 654). In an intervention for all students, Palfai et al. (56) reported a small effect reduction in any drinking. In the study enrolling athletes only (53), the intervention was associated with a larger decrease in peak eBAC at 6-month follow-up compared to that of control. Other multi-component intervention studies did not yield substantial evidence supporting their efficacy.

Though all studies included some form of personalized feedback, other intervention components differed. Among multi-component intervention trials included in this review (*k* = 12), it was suggested that inclusion of multiple intervention components may be associated with stronger evidence of efficacy. Of the seven multi-component intervention studies that included personalized feedback, protective behavioral strategies, facts about alcohol, and resources for alcohol reduction, five reported at least one effect size estimate of 0.20 or greater (19, 48, 50, 51, 56), while only two out of five multi-component studies that did not include all components reported an effect size of this level (49, 53).

## Discussion

Overall, the present review yielded some evidence supporting the efficacy of very-brief, web-based interventions for college students in the reduction of alcohol consumption, though effect sizes tended to be small and several studies (*k* = 6) reported no intervention effects of 0.20 or greater compared to control. It is also worth noting that RCTs included in this review varied with regard to the alcohol-related outcomes they reported and the outcomes associated with substantial intervention effects also varied across trials. Other prior reviews and meta-analyses [e.g., Ref. (15, 16)] have found at most only small effect size advantages for alcohol reduction brief interventions compared to control conditions among young people. The present review did not yield evidence that very-brief, web-based interventions are efficacious in reducing alcohol-related problems among college students. Only one out of the 10 studies reporting alcohol-problems outcomes yielded any outcome with an effect size of 0.20 or greater. These results are in accordance with a recent meta-analysis of motivational interviewing-based interventions for young drinkers, which found an overall lack of effect in reducing consequences (15).

Given the small number of studies in this review, our ability to detect relationships between key study and intervention characteristics and effect size estimates for alcohol outcomes was necessarily limited. However, the oldest paper included in this review was published in 2008. Thus, very-brief, web-based alcohol use reduction interventions for college students is a relatively new area of research attention, which we expect to continue in the future. In addition to the potential public health benefit of expanded research in this area, an increase in the number of published reports should allow for a determination of which study and intervention characteristics are associated with enhanced efficacy. Efforts are needed to optimize intervention content to enhance efficacy for as many young adult drinkers as possible. Mismatches between intervention type and key individual characteristics have been linked to negative outcomes (60). While efficacious overall, effect sizes of brief interventions tend to be small and less efficacious for the highest-risk drinkers (8), thus efforts are needed to enhance intervention efficacy, in particular, among the most severe drinkers.

In terms of intervention content, it was telling that all eligible RCTs tested some form of personalized alcohol feedback. The goal of personalized feedback is “to activate existing self-regulatory processes, in part through highlighting discrepancies between the individual’s current behavior and his or her goals, values or desired state of being” [(61), p. 285]. Highlighting discrepancy to spur positive behavior change is consonant with motivational interviewing principles (62). Multiple meta-analyses have identified personalized feedback as an efficacious component of brief interventions (8, 10). Furthermore, a recent review of 41 studies confirmed the reliable efficacy of personalized feedback in reducing drinking among college students (13). While a focus on personalized feedback is well-founded empirically, again, currently available brief interventions tend to have small effect sizes, thus novel intervention approaches for young adult drinkers are needed (63).

The present review yielded results suggesting that very-brief web-based interventions focused on personalized normative feedback and those including multiple intervention components have evidence of efficacy. Regarding the latter type of intervention, the work of two research groups stands out due to publication of multiple reports suggesting the efficacy of their interventions with Bewick et al. (19, 48) reporting decreases in units per occasion and Kypri et al. (50, 51) reporting decreased heavy drinking. However, the Bewick et al. trials raised concerns regarding risk of performance and attrition bias (Table 2). In two recent, very large RCTs, the Kypri et al. intervention had stronger evidence of efficacy among a particularly high-risk indigenous population (2013) than among at-risk drinking students in general (2014). A prior RCT, however, conducted among a general population of at-risk drinking students yielded evidence of efficacy (50).

Several other studies also raised concerns regarding performance and attrition bias. Assessment-only control inherently raises concerns as participants randomized to such a condition spend notably less time in study participation than those randomized to an intervention condition. Colleges are typically close-knit communities, making it relatively easy for students to communicate with one another about a research study. Steps can be taken to offset this risk. For instance, Kypri et al. (50–52) couched their studies as series of surveys rather than an intervention study. Alternatively, in other studies, participants were informed that they were to be randomized to a study condition, which may or may not entail provision of additional information about campus drinking patterns. While it is impossible to blind participants to study condition in the same way as in a medication RCT, efforts should be taken to avoid conveying to participants that they may be in a control condition, in order to avoid biasing results. Multiple studies had high attrition rates while others reported differences in attrition rates across conditions or failure to account for missing data analytically, all of which could bias results. Overall, high risk of bias was suggested for at least one criterion in the majority of studies in this review.

The present review had a number of limitations, including the small number of studies. In addition, effect size estimates that we made based upon means and standard deviations reported in publications may not be ideal as most alcohol outcome data are best thought of as being in count rather than truly continuous format [see Ref. (26, 31)]. Also, methodological differences across studies, notably differences in follow-up time-points, make comparisons across studies challenging. Our approach of tallying RCTs based on key distinctions (Table 2), which followed Larimer and Cronce’s approach (11, 12, 41), does not account for differences in sample sizes. However, we considered sample sizes of RCTs in our narrative description of the results. In addition, sample sizes are reported for each RCT in Table 1 so that readers may take this information into consideration when evaluating the results of each trial. Weighting for sample size is one advantage of meta-analysis methods, however for reasons stated above, we believed that a meta-analysis of this literature was premature.

In conclusion, the present review demonstrated that there is some evidence to support the efficacy of very-brief, web-based interventions among college students for alcohol use reduction, though the outcomes indicating efficacy varied from study to study and there was not substantial evidence supporting efficacy in ameliorating alcohol-related problems. Risk of bias is also a consideration as 10 out of the 15 studies included in this review raised a high degree of concern on at least one criterion. Future research is needed to test enhancements to very-brief, web-based interventions that feature personalized feedback and to determine for which types of college student drinkers these interventions are most efficacious. In addition, future studies are needed to test novel, very-brief, web-based interventions featuring approaches other than personalized feedback. Very-brief, web-based interventions are worth pursuing given their strong inherent advantages of convenience, ease of dissemination, and college students’ preference for this type of intervention.

## Conflict of Interest Statement

This research was conducted in the absence of any commercial or financial relationships that could be construed as a potential conflict of interest.
